# Safety of Stromal Vascular Fraction Cell Therapy for Chronic Kidney Disease of Unknown Cause (Mesoamerican Nephropathy)

**DOI:** 10.1093/stcltm/szac080

**Published:** 2022-12-21

**Authors:** Michael H Carstens, Nelson García, Sreedhar Mandayam, Biruh Workeneh, Indiana Pastora, Carlos Calderón, Kenneth A Bertram, Diego Correa

**Affiliations:** Department of Surgery, Universidad Nacional Autónoma de Nicaragua, León, Nicaragua; Hospital Vivian Pellas, Managua, Nicaragua; Wake Forest University Institute for Regenerative Medicine, Winston-Salem, NC, USA; Department of Medicine, Universidad Nacional Autónoma de Nicaragua, León, Nicaragua; Nephrology section, Ministerio de Salud, República de Nicaragua; Department of Nephrology, University of Texas MD Anderson Cancer Center, Houston, TX, USA; Department of Nephrology, University of Texas MD Anderson Cancer Center, Houston, TX, USA; Department of Medicine, Universidad Nacional Autónoma de Nicaragua, León, Nicaragua; Department of Cardiology, Hospital San Juan de Dios, San José, Costa Rica; Wake Forest University Institute for Regenerative Medicine, Winston-Salem, NC, USA; Diabetes Research Institute & Cell Transplant Center, University of Miami Miller School of Medicine, Miami, FL, USA; Diabetes Research Institute Federation, Hollywood, FL 33021, USA

**Keywords:** chronic kidney disease of unknown cause (CKDu), Mesoamerican nephropathy, neovascularization, stromal vascular fraction (SVF), cell-based therapy, safety

## Abstract

Chronic kidney disease of unknown cause (CKDu), also known as Mesoamerican nephropathy, typically presents as an ischemic nephropathy with chronic tubulointerstitial fibrosis in normotensive patients, rapidly progressing to kidney failure. In this first-in-human, open-label, safety study, we followed 18 patients with CKDu (stages 3-5) for 36 months after receiving a single infusion of angiogenic/anti-fibrotic autologous adipose-derived stromal vascular fraction (SVF) cells into their kidneys bilaterally via renal artery catheterization. SVF therapy was safe and well tolerated. There were no SVF-related serious adverse events and no procedural complications. Color Doppler evaluation at 2 months demonstrated increased perfusion to the interlobar and/or arcuate artery levels in each kidney evaluated (36/36) with a reduction in resistance index at the hilar artery (35/36) kidneys. Beyond 12 months, patients with initial eGFR <30 mL/minute/1.73 m^2^ deteriorated, whereas those ≥30 mL/minute/1.73 m^2^ further sustained their renal function, suggesting a possible renal protective effect in that group.

## Graphical Abstract

**Graphical Abstract:**
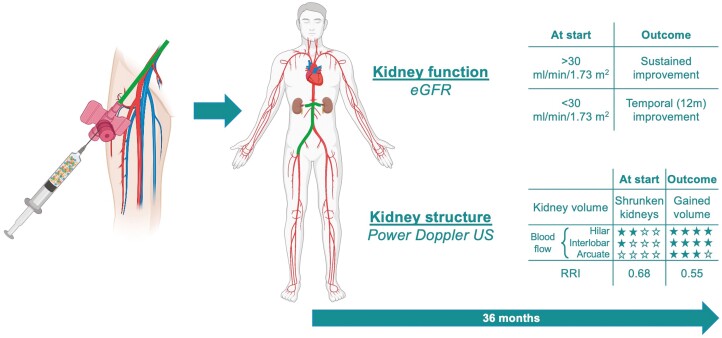

**Lessons Learned**

- Stromal vascular fraction cells (SVF) can be safely infused into the renal artery.
- In patients with Mesoamerican nephropathy/chronic kidney disease of unknown cause (CKDU), intra-arterial SVF cell induces changes in the renal vasculature characterized by expansion of blood distribution and reduction in the renal artery resistance index.
- SVF cells infused into CKDu patients with early stage disease (eGFR >30) appear to demonstrate a renoprotective effect.

**Significance Statement**

Mesoamerican nephropathy is a rapidly progressive disease of unknown cause which is uniformly fatal. In this first-in-man study, SVF cells processed at the point of care can be safely injected into the renal artery. A subset of patients remain without renal deterioration 36 months after infusion. To our knowledge, no other therapy has been demonstrated to have an effect in slowing or halting the progression of this disease

## Introduction

Chronic kidney disease of unknown cause (CKDu), formerly known as Mesoamerican nephropathy, is an aggressive variant of chronic kidney disease first described in individuals in the coastal regions of Central America, including Nicaragua.^1,2^ Affected patients are often young, non-diabetic, normotensive, asymptomatic, male agricultural workers. They may present with constitutional symptoms, eg, fatigue, or they may have an abnormal urinalysis detected as part of routine healthcare if they work on a sugar plantation. Laboratory evaluation typically shows an elevated serum creatinine, reduced estimated glomerular filtration rate (eGFR), and an urinalysis with minimal or no non-nephrotic proteinuria, hyperuricemia, hyponatremia, and hypokalemia.^3^ The time to progression to end-stage disease is still not well established, but by some estimates, renal function declines 3.8-4.4 mL/minute/1.73 m^2^ per year.^4^ At the time of diagnosis, most patients are already in chronic kidney disease (CKD) stages 3-4, suggesting a rapid decline in kidney function. The stages of CKD, regardless of etiology, are defined by eGFR, with normal defined as ≥120, stage 1 is 119-90; stage 2 is 89-60; stage 3 is 59-30; stage 4 is 29-15; and stage 5 < 15.^5^ Patient entry into the Nicaragua health system for CKDu shows considerable variation, often being diagnosed late in the disease. Of 36 patients treated at the renal dialysis unit of Hospital Escuela Oscar Danilo Rosales Argüello (University Hospital, Leon) in 2021, 13 (36%) initially presented in stage 5, 16 (44%) were in stage 4 and (19%) were in stage 3. Length of time of the disease is likewise uncertain but in the above dialysis group, 80% had been diagnosed with CKDu for less 5 years while 20% had a longer disease course (Carstens, 2021, unpublished).

Multiple investigations in the etiology of CKDu have examined infectious causes, agricultural pesticides, and genetic mutations—yielding conflicting data. Most recently, heavy metals have been implicated.^6^ The causes of CKDu therefore may be multifactorial,^7-9^ aggravated by repetitive episodes of dehydration-related acute kidney injury. Pathologically, acute tubulointerstitial nephritis^10^ progresses to chronic interstitial disease with tubular atrophy and thickened basement membrane, glomerular hypertrophy (by some reports), and ischemia leading to interstitial fibrosis and glomerulosclerosis (by some reports).^11-13^

Management of CKDu in Nicaragua is primarily focused on slowing disease progression by monitoring patients for diet compliance; management of cardiac risks; avoidance of nephro-toxic medications; and management of hyperuricemia. No effective therapy exists to halt the structural and functional alterations in CKDu. This disease is associated with high rates of mortality in Central American countries as dialysis is often not available.

Histopathologic findings suggest that an effective treatment would need to block the acute and chronic inflammatory/fibrotic events. The observation of ischemic changes in affected glomeruli, such as wrinkling, suggests the possibility of compromised renal perfusion. Therefore, therapeutic interventions that promote local vasodilation and angiogenesis could be beneficial, with a goal of repair and recovery of glomeruli and tubules.

One such intervention could be stem cell therapy. Multipotent stem cells [now called mesenchymal stem/stromal cells (MSC)], originally found within the bone marrow, are capable of differentiating into various cell types, which raised the possibility that stromal cells could be used to treat multiple diseases.^14^ In fact, MSC from other tissue sources (adipose, placenta, umbilical cord) have been shown to recognize sites of injury, “sense” the environment, and selectively respond through multiple pathways to support the remodeling/repair of the injured tissues. MSC are now thought to primarily work via a paracrine pathway (as opposed to cell differentiation). The secretion of various cytokines (eg, IL-6), growth factors [eg, vascular endothelial growth factor (VEGF)], and other immune and fibrosis mediators have been shown to mitigate existing tissue and cellular damage, and in some pathological disease states, to stimulate local angiogenesis, cause vasodilation, and heal the damaged tissue.^14^

While early studies investigated the role of stem cells in kidney repair by giving bone marrow MSCs directly into the parenchyma of the kidney, one of the least complicated forms of cellular intervention is to administer adipose-derived MSCs (ADMSC) via renal artery infusion. Renal artery perfusion has been shown to be an excellent method for delivering stems cells to aid kidney repair in experimental models.^15^ Using this method, ADMSCs have been shown to improve vascular flow and repair underlying tissue damage in ischemic,^16^ inflammatory,^17,18^ and obstructive-induced^15,18^ nephropathy animal models. We hypothesized that ADMSC-associated paracrine proteins would stimulate vascular formation, produce a remodeling of the extracellular matrix, and modulate inflammatory responses. Thus, there is sufficient experimental evidence to warrant a pilot study in humans to test the feasibility and potential benefit of ADMSC therapy in CKDu.

One method to obtain ADMSC at point of care and perform a same day cell infusion is through the use of liposuction, followed by collagenase treatment and washing of the lipoaspirate. The resulting cell mixture is called stromal vascular fraction (SVF) and contains numerous therapeutically-active cellular components. Brown et al. have analyzed SVF composition using cell lineage markers and fluorescence-activated cell sorting (FACS) and have documented the presence of ADMSCs, pericytes, endothelial cells, adipose and hematopoietic progenitors, and monocytes.^19^ Advantages of SVF are: large numbers of ADMSCs (500× greater than from bone marrow), easy access from the patient’s own fat (thus avoiding any immune complications); rapid processing time (within 3 hours), thus avoiding the time and cost of cell culture. In short, SVF cells can be transplanted into the patient’s kidneys as a same-day procedure at the point of care.^20,21^ SVF has been shown to be safe in other inflammatory conditions, such as osteoarthritis, with some early evidence of efficacy, as reviewed by Garza et al.^22^

The purpose of this first-in-human, open-label study was to determine feasibility and safety, and to gain potential evidence of clinical utility, of a single infusion of autologous SVF cells administered via renal artery catheterization in patients with stages 3-5 CKDu. The issues of feasibility for this trial included the ability to identify suitable patients, the willingness of patients to participate in a clinical trial, and the ability of patients to make all the required visits. The CKDu-affected population in Nicaragua tends to a physical-labor, rural working-class socio-economic background. Nicaragua is a small country of 6.5 millions with a centralized public medical system and a limited research capability; therefore, for purposes of this study, “feasibility” included the ability to collect all the required data. Critical issues for safety included complications from the invasive procedures of lipoaspiration and intrarenal artery perfusion (infection, bleeding, artery laceration, infection, bleeding, thromboembolism), and/or complications of the SVF cells (embolism, infection, acute or chronic renal insult, hypertension, neoplasia). Potential post-treatment efficacy was evaluated in terms of changes in vascular flow and renal function as outlined in Methods.

We report herein the 36-month follow-up of 18 CKDu patients (36 kidneys) treated with SVF with regard to feasibility, safety, and potential clinical utility.

## Methods

### Ethics

This study (clinicaltrials.gov number NCT05154591) was approved by the Medical Ethics Committee of Universidad Nacional Autónoma de Nicaragua (UNAN)-León and sponsored by the Ministry of Health of Nicaragua (MINSA). Human subject procedures were in accordance with the UNAN-León requirements and the Helsinki Declarations of 2000. Informed consent was obtained using the standards of MINSA and the World Health Organization including consent to publish in all formats. Under the requirements of MINSA, the design of clinical studies in life-threatening diseases such as CKDu cannot include placebo treatments.

### Patient Selection and Pre-SVF Treatment Evaluations

Patients identified at community health centers were screened by the staff nephrologist; and confirmatory laboratory studies of serum and urine were performed. Eighteen male agricultural workers, 29-55 years of age (45.2 ± 8.5 mean ± SD years), were recruited at UNAN-Leon with a diagnosis of CKDu and no other co-morbidities. Characteristics of these patients upon presentation are summarized in Table 1 (Results section). Patients were staged based on eGFR, calculated by the CKD-EPI equation, as it factors in for age, gender, race, and creatinine threshold of 0.9 [Levey].^23^ eGFR = 141 × (Scr/0.9)^−0.441^ × (0.993)^Age^. There were 2 patients in stage 3a (eGFR 59-45), 6 patients in stage 3b (eGFR 44-30), 7 patients in stage 4 and 3 patients in stage 5. Stages 4 and 5 are considered CKDu advanced disease.^5^ Recruited study patients were then followed for 3 months prior to SVF treatment to assess the stability in renal function. Standard of care treatment during the 3-month run-in period consisted of monthly follow-up checks to ensure compliance with therapeutic goals: control of blood pressure, anemia, hyperuricemia, alterations in acid-base status, and electrolytes.

**Table 1.** SVF-treated subject characteristics and safety.

Subject number	Age entry into study	CKD stage at study entry	# SVF cells infused Xe6	SAE	Study-attributable SAE
1	52	3a	120.6	None	No
2	39	3a	189.6	None	No
3	49	3b	35.3	None	No
4	51	3b	142.7	None	No
5	29	3b	44.3	None	No
6	46	3b	74.9	None	No
7	51	3b	60.1	None	No
8	52	3b	84.0	None	No
9	52	4	66.6	None	No
10	34	4	31.7	None	No
11	39	4	60.6	None	No
12	49	4	50.9	None	No
13	36	4	66.4	Dialysis indicated	No
14	50	4	84.8	Dialysis indicated	No
15	44	4	78.1	Dialysis indicated	No
16	44	5	99.2	Death	No
17	30	5	81.6	Death	No
18	55	5	51.2	Death	No

All subjects were male. Deaths occurred in two stage 5 patients, who died of deterioration of their renal failure, and one stage 5 patient, who died of a complication of intracerebral hemorrhage. No deaths were attributable to study invasive procedures or SVF cell infusion. Abbreviations: CKD, chronic kidney disease, SAE, serious adverse events; SVF, stromal vascular fraction.

### Historical Comparison Group

As stated under Ethics, the design of clinical studies in life-threatening diseases cannot include placebo treatments. Therefore, a historical CDKu cohort of 12 male patients was selected with similar age and CKD stages to serve as a comparison group. These patients ranged in age 31 to 68 years (48.5 ± 11.2 mean ±SD) with 6 patients starting at stage 3 and 6 patients starting at stage 4. Their records reflected that they received comparable Nicaragua standard of care available (2005-2021). A clinical profile of the patients in the historical cohort is presented in Table 2 (Results section).

**Table 2.** Historical comparison cohort.

Subject Number	Age at diagnosis	eGFR time of diagnosis	CKD stage at diagnosis	Time (months) between diagnosis and need for dialysis (eGFR <15)
1	32	45	3a	24
2	42	43	3b	60
3	46	41	3b	14
4	31	41	3b	28
5	51	39	3b	04
6	50	38	3b	60
7	68	28	4	11
8	51	26	4	22
9	59	26	4	24
10	59	24	4	24
11	59	21	4	39
12	47	18	4	04

All subjects were male. Progression to end-stage renal disease (defined as reaching an eGFR of <15 mL/minute/1.73 m^2^) was calculated in months. Abbreviations: CKD, chronic kidney disease; eGFR, estimated glomerular filtration rate.

### SVF Preparation and Administration

#### Lipoaspiration

**Figure 1.**
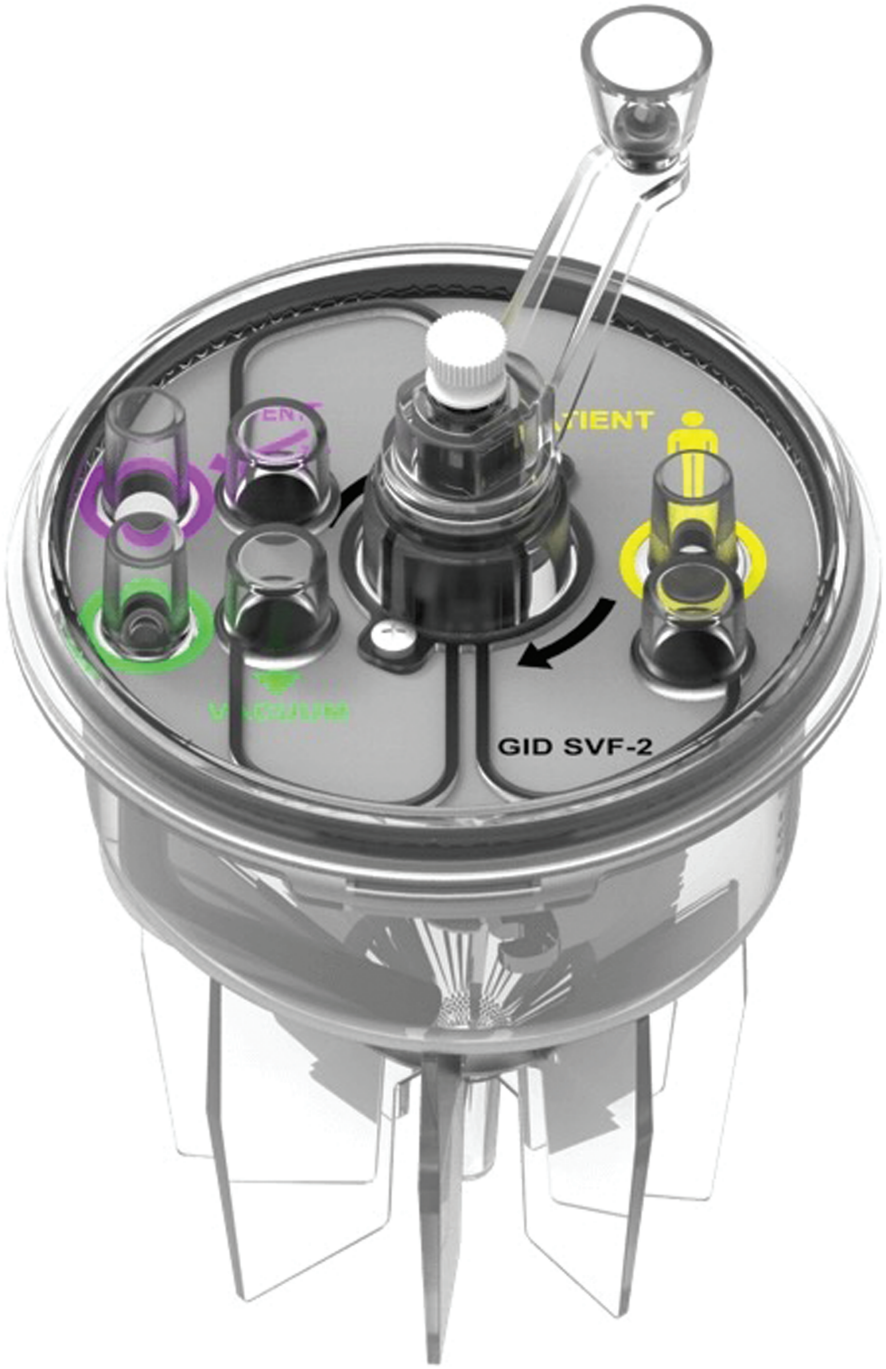
Graphic explaining SVF cell processing technology using the GID Bio SVF-2 device. Collection and digestion of lipoaspirate take place within a closed-system sterile device The GID SVF-2 device (GID Bio, Louisville, Colorado) is a single-use, sterile, closed-system for the collection and collagenase processing of lipoaspirate within a chamber located above a 400 nm mesh filter. Subsequent centrifugation at 600*g* drives the SVF cells through the mesh into an aspiration chanber. Collagen fibers and any remaining adipocytes (diameter ≥ 400 nm remain entrapped by the mesh. Rotating paddles above the mesh cull out remaining cells 2 additional centrifugations. The SVF is then accessed by passing a #14 needle although the white diaphragm, perforating the mesh and entering the reception chamber. After aspiration and re-suspended, and a cell count, the SVF cells are administered. For colour figure refer to online version. For a detailed description, see Brown et al.^23^

Prior to surgical intervention, coagulation profiles and serum chemistries were performed. Under general anesthesia, the liposuction site (abdomen) was infiltrated with 250 mL of standard wetting solution consisting of 1000 mL of Ringer’s lactate to which was added 50 mL of 1% lidocaine with epinephrine. After waiting 15 minutes for the hemostatic effect of epinephrine to take effect, 200-300 mL of lipoaspirate were collected using a 3-mm blunt-tip Mercedes cannula. The lipoaspirate was drawn directly into a sterile processing canister (GID SVF-1, Louisville, CO, USA; Fig. 1).

#### Processing of SVF

The closed-system method of SVF processing has been previously described by Carstens et al.^24^ In summary, the lipoaspirate tissue was washed, dissociated for 50 minutes with collagenase (Worthington CLS-1, Lakewood, NJ, USA) at a concentration of 200 collagen digestion units (CDU)/mL of total volume, followed by the addition of 40 mL human albumin to stop the reaction. SVF cells were separated via centrifugation for 10 minutes at 800*g*. The cell pellet was extracted and resuspended in Hartmann solution; with an aliquot (10 µL) removed for counting and viability assessment of resulting total nucleated cells through an image cytometer (ADAM MC, Portsmouth, NH, USA). The process of isolation and preparation of SVF took an average of 3 hours. As SVF from multiple individuals has been previously well characterized (as described above), only cell number and viability were assessed prior to renal artery infusion.^19^

#### SVF Infusion

The number of SVF cells infused varied among patients based upon the total number of SVF cells obtained from each individual’s adipose tissue (Table 1). Femoral artery cannulation under fluoroscopy permitted advancement of a 100-cm balloon-tip catheter into each renal artery, with position confirmation using 1 mL of OptiRay 320 contrast diluted 1:4 with Hartmann solution. SVF cells admixed with 200 mL of warmed Hartman’s were then infused using a DRE pump (DRE, Louisville, KY) over 15 minutes with constant agitation. Each subject kidney received one-half of their total SVF cells. That is, both kidneys were treated with SVF. All of the procedures for a given subject were completed on the same day.

### Feasibility Evaluation

Feasibility was assessed by recruitment difficulty, percent of appointments or contacts missed, and completeness of data collection.

### Safety Monitoring

Subjects were followed in the hospital for immediate adverse events related with the lipoaspiration procedure (eg, bleeding); renal artery catheterization (eg, renal embolism, hemorrhage); and SVF cell infusion (eg, anaphylactic reaction, fever, hemodynamic alterations, and acute kidney ischemia) for 24 hours. After discharge, patients were interviewed for adverse events daily by phone for 1 week, then weekly for a month.

Subjects followed up in clinic for history, medication review, and physical exams at 2-, 4-, 6-, 12, 18-, 24-, 30-, and 36-months post-SVF treatment. Laboratory visits conducted during follow-up visits included: complete blood count, serum chemistry, urinalysis, and urine chemistry. All subjects had direct access to the clinical coordinating team by cell phone if needed. Follow-up clinic visits are summarized in Supplementary Table S1 and S2.

### Clinical Utility Assessment

Renal ultrasound provides qualitative evidence of distribution of renal blood flow as evidenced by changes in the color doppler signal as it progresses from the large hilar vessels toward smaller arcades deeper into the kidney. Thus, if renal perfusion improves in response to therapy, the color doppler signal should be evident from the hilum into the interlobar circulation and finally the arcuate arterial arcade located at the cortico-medullary junction. Another measurement of renal vascular flow is the renal resistance index (0-1.0) which reflects the total cross-sectional area of the renal artery network. These measurements were carried out at the level of the hilar artery.^21,22,25,26^ Post-treatment clinical utility was also assessed in terms of renal function as determined by glomerular filtration rate (eGFR).

All 36 individual kidneys were assessed by a single radiologist using a Toshiba Canon Aplio 300 CV ultrasound machine for changes in distribution of blood flow. The radiologist was not blinded as all subjects were treated and results were needed in real time as part of safety evaluations. Renal resistive index (RRI) calculated from serial measurements of the arterial wave form, noting the difference in the systolic and diastolic velocity peaks. As reported in the literature, an RRI value of >0.70 is considered abnormal. A change in RRI value (Δ) ≥ 0.02 between measurements is clinically significant.^22,25,26^ Serum creatinine levels were obtained pre-treatment and then at each follow-up; these were used to calculate eGFR (as explained above). Urinary protein and electrolytes were monitored by serial urinalysis.

### Statistical Analysis

Paired *t*-tests were used to compare pre- and post-therapy changes in renal volume and hilar RRI. Statistical analyses were performed using GraphPad Prism version 8.1.0 (San Diego, CA), and significance level set up at *P* < .05.

## Results

### Cell Product Dose

Lipoaspirate generated 243.8 ± 79.5 g of adipose tissue per patient, yielding an average of 79 ± 39.4 × 10^6^ viable cells (83.6 ± 6.3% viability) for infusion (Table 1). As an optimum treatment dose for this disease state was unknown, each patient received the number of cells produced from their individual lipoaspirates. These individual doses all exceeded a minimum total of 35 × 10^6^ SVF cells based upon previously published experience by our team in patients with lower extremity arterial insufficiency.^24^ Neither the total number of SVF cells obtained, nor the cell viability, were related to CKDu stage at entry.

### Feasibility

Study personnel were easily able to identify suitable patients. Given the relentless and fatal nature of the disease, patients were eager to participate in the study. Although no efficacy outcomes were promised, of 19 patients screened, 18 were enrolled. The study team was able to maintain contact with all the subjects and all subjects completed the study, apart from the below described non-SVF-related deaths. The study team was able to collect the protocol-specified data in a timely and complete manner.

### Safety Assessment

Three periods were monitored for safety: (1) acute events related to the invasive procedures, (2) acute post-SFV infusion systemic reactions, and (3) long-term clinical events after SVF infusion. Lipoaspirates, transaortic catheterization of the renal arteries, and SVF cell infusions were all performed with no adverse events attributed to the procedures. No allergic reactions were noted.

Ten patients entered the study in advanced stages of CKDu. Of those, 3 patients in stage 5 died at 20, 24, and 28 months, respectively. The first patient succumbed to a cerebral hemorrhage at 20 months, while the other 2 patients in stage 5 died from further deterioration in their renal function. Review of these cases by the chief MINSA nephrologist (not a member of the study team) revealed no treatment-related mortality given the underlying stage of their disease. Progressive declines in renal function in stage 4 and 5 CKDu patients were consistent with known disease course. No other chronic adverse events (hypertension or evidence of neoplasia within the kidney parenchyma) occurred during the 36 months post-treatment.

### Potential Clinical Utility

#### Renal Volume

**Figure 2.**
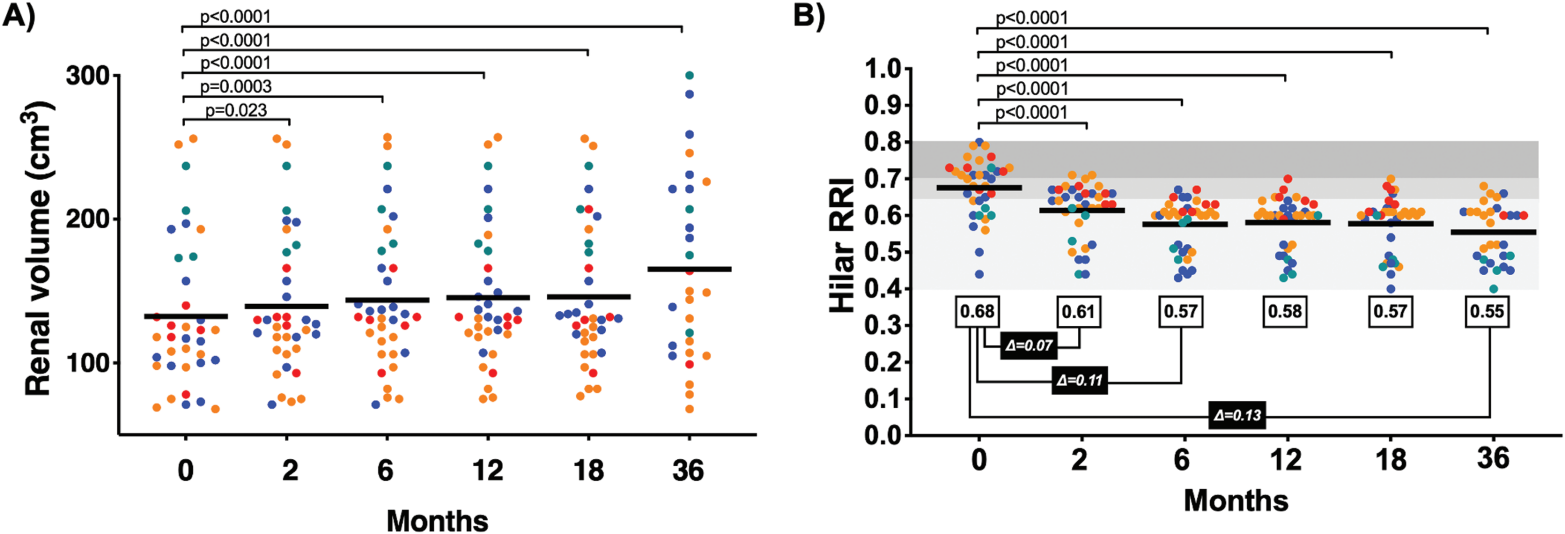
Renal volume and renal artery resistance index measured over time.(A) Renal volume (cm3) per individual patient, by chronic kidney disease stage (color dots: Stage 3a – green; 3b – blue; 4 – orange; and 5 – red), from pre-treatment (time 0) to 36 months post-treatment. Mean volume of total kidneys represented by solid bar at each time point. (B) Renal artery resistance index at the hilum (RRI) per individual patient, by chronic kidney disease stage (color dots: Stage 3a – green; 3b – blue; 4 – orange; and 5 – red), from pre-treatment (time 0) to 36 months post-treatment. Mean RRI of all kidneys represented by solid bar at each time point (for colour figure refer to online version).

Kidney size is known to decrease in length and volume as chronic kidney disease progresses, and recovery of kidney volume in CKD is exceptionally rare. We therefore measured kidney volume as one surrogate marker for kidney repair. While acknowledging a small sample size, non-blinded evaluations, and a single radiologist as reader, the ultrasound studies over 36 months suggest a trend toward an increase in kidney volume after treatment with SVF (Fig. 2A). At 12 months, a total of 26/36 kidneys were larger compared with baseline. At 36 months (taking into consideration the 3 patients deceased) 24/30 kidneys remained larger than baseline.

#### Resistance Index, Hilar Artery

CKD is also associated with a high resistance index and decreased renal blood flow due to progressive injury and vasoconstriction of the renal vascular system. Therefore, measuring renal resistance indices can represent another surrogate test for kidney repair and improvement. At baseline, the study patient group mean hilar RRI was 0.68, primarily driven by patients with advanced disease (>0.70). Two months post-therapy, 31/36 kidneys showed a reduction (improvement) in RRI to an average of 0.61 (a Δ = 0.07 units). By 6 months, 34/36 kidneys responded, as the group RRI further declined (improved) to 0.57 (Δ = 0.11), which stabilized during the rest of the study until 36 months where the final average (subtracting the 3 patients who died) was calculated as 0.55 (Δ = 0.13) (Fig. 2B).

#### Renal Function

**Figure 3.**
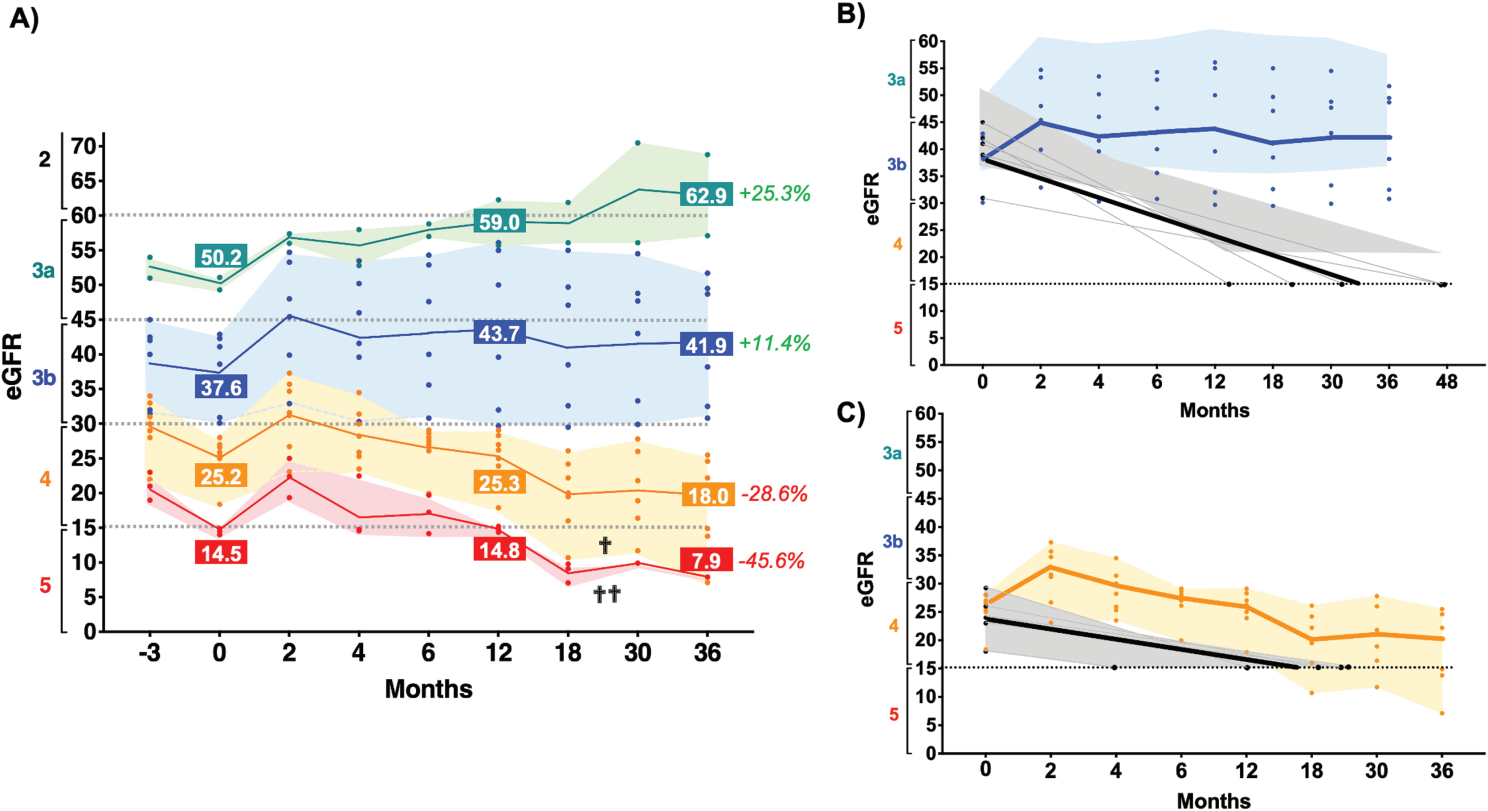
Kidney function assessment (eGFR) measured over time. (A) Overall renal function. eGFR (grouped by chronic kidney disease category) per individual patient (color dots) from 3 months pre-treatment to 36 months post-treatment and the average per stage-group (solid color line), for the 4 different patient groups represented in shaded colors (3a – green; 3b – blue; 4 – orange; and 5 – red). Overall percentage improvement (+, green) or deterioration (-, red) in eGFR (renal function) by patient group at 36 months. (B) Renal function (eGFR) over 36 months for the 6 SVF treatment patients that were Stage 3b at the initiation of the study compared to untreated Stage 3b historical controls. No SVF treated patients declined in renal function, thus avoiding the need for renal dialysis (Blue field). In contrast, the untreated historical control Stage 3b group (5 patients) had a decline in renal function, requiring dialysis (eGFR <15 ML/minute) between 4-48 months (mean – 33 months) after diagnosis (Gray field). (C) Renal function (eGFR) over 36 months for the 7 SVF treatment patients that were Stage 4 at the initiation of the study compared to untreated Stage 4 historical controls. While the SVF treated patients overall demonstrated a decline in renal function, only 3 needed renal dialysis (range of 18 to 36 months post treatment; Orange field). The untreated historical control Stage 4 group (5 patients) had a more drastic decline in renal function, requiring dialysis (eGFR <15 ML/minute) between 4-24 months (mean - 17 months) after diagnosis (Gray field) (for colour figure refer to online version).

A decline in renal function (eGFR) was evident in all patients during the pre-therapy period (3 months) and was markedly more pronounced in advanced stages (Fig. 3A). At 2 months post-therapy, rapid improvement in eGFR was observed across all stages. Of note, this early clinical responses were observed in all 18 patients, independent of the final total cell dose received (Pearson *r* = .4609; CI −0.007594 to 0.7635; *P* = .054). At 12 months, the improvement in function (eGFR) was maintained in patients in stages 3a and 3b, while it was progressively lost in patients who had advanced disease at the time of SVF treatment. At 36 months, patients with initial eGFR > 30 (stages 3a and 3b) maintained and further improved renal function, gaining 25.1% (stage 3a) and 12.4% (stage 3a) function. No SVF-treated stage 3a or 3b patients required dialysis out to 36 months of follow-up. In contrast, the 5 stage 3b subjects in the matched historical cohort, who started with eGFRs ranging between 30 and 44 mL/minute, reached an eGFR of ≤15 and became dialysis candidates at a mean of 33 months (range 14-48 months) (Table 2; Fig. 3B).

SVF-treated patients with initial eGFRs <30 continued to deteriorate, losing 29.2% and 45.3% of renal function at 36 months for stages 4 and 5, respectively. Two of the stage 4 CKDu patients became candidates for dialysis by 18 months and a third stage 4 patient became a candidate for dialysis by 36 months. For the stage 5 SVF-treated CKDu patients, all became candidates for dialysis by 12 months. The historical cohort with 6 stage 4 CKDu patients, who started with a mean eGFR of 24, declined to an eGFR of 15, becoming candidates for dialysis between 4 and 24 months. The SVF-treated stage 4 patients appeared to be delayed in needing dialysis compared with the historical stage 4 cohort (Fig. 3C).

## Discussion

CKDu is an aggressive nephropathy of unknown etiology and is often fatal in resource-poor countries that have limited access to dialysis care. This first-in-human study shows that administration of adipose derived, autologous SVF cells via renal artery catheterization is feasible and safe. In addition, this study provides potential evidence that intra-arterial SVF treatment may result in improved kidney function over a sustained period of time, principally in early stages of the disease.

Recent pathology studies suggest that CKDu begins with an acute insult to the kidney, potentially due to environmental causes (heat stress, dehydration, toxic chemicals), drugs, or infectious agents. The initial pathology in most patients who subsequently meet the criteria for CKDu reveals tubulointerstitial nephritis with varying degrees of acute tubular injury, interstitial edema, interstitial immune cellular infiltrates, and early fibrosis.^10^ Over time the disease seems to evolve to severe tubular atrophy, thickened tubular basement membranes and fibrosis, with little evidence of immune cells. There remains a controversy as to the degree of concurrent glomeruli damage during the disease and whether this is a primary or secondary process. However, the wrinkling of glomerular basement membrane observed in many patients suggests ischemia and a vascular component to the kidney injury in addition to tubular damage and local inflammation. These early disease renal pathology findings highlight the importance of a therapeutic intervention that can reverse the pro-inflammatory and fibrotic states and stimulate angiogenesis. The pathologic findings in late disease may reflect a severe “burnt-out” state with extensive fibrosis, decreased cells, and decreased vascular flow resulting in kidney tissue damage that may be beyond repair.^13^

The preliminary clinical results of this study appear to correlate with the above pathology studies, that is, the earlier CKDu patients (stages 3a and 3b) had a response to SVF that could reflect the known properties of SVF which include anti-inflammatory and anti-fibrotic properties, as well as the potential for tissue healing. These earlier stage patients had an increase in kidney volume which could be consistent with tissue healing and decreased fibrosis. They also demonstrated improved hilar RRI consistent with improved blood flow. Enhanced circulation could be related an increase in local formation of new blood vessels, perhaps in response to the known capability of MSC to produce and secrete vascular endothelial growth factor (VEGF). In contrast, the stages 4 and 5 patients had only a temporary improvement to the SVF therapy, consistent with a more “burnt-out” tissue state.

Textor, Saad, and team looked at the safety, dose, and early evidence of efficacy of autologous *cultured* ADMSC to treat kidney disease accompanying atherosclerotic vascular disease in which microvascular injury is prominent.^27^ They treated 39 patients medically to optimize renal function and then treated 6 patients with low dose (1 × 10^5^ cells/kg) autologous cultured adipose MSC via unilateral renal artery infusion; another 7 patients with medium dose (2.5 × 10^5^ cells/kg) and 6 patients with high dose (5 × 10^5^ cells/kg). Eighteen patients did not receive cells (medical therapy controls). They were able to follow the response to therapy in the patients by GFR, MRI imaging of the kidneys, and obtaining blood from the renal vein pre-treatment and 3 months after cell therapy. Results in the cultured ADMSC-treated kidneys showed a modest change in GFR (most evident in the highest dose); improved vascular flow; and decreased pro-inflammatory cytokines in the renal vein blood after treatment compared to baseline, while the 18 patients controls had no changes in these functions. Interestingly, for a given individual patient, the untreated (contralateral kidney) improved in renal blood flow to a similar degree as observed in the treated kidney, suggesting systemic paracrine mediators.^28,29^

Thus, the relevant physiologic mechanisms by which SVF might improve CKDu would seem to be aligned with the same mechanisms suggested by the beneficial treatment of atherosclerotic vascular renal disease by cultured adipose MSCs, that is, a decreased pro-inflammatory state, decreased fibrosis, and improved vascular flow. These changes in the underlying renal disease state are most likely due to the paracrine mediators released by the infused adipose stem/stromal cells upon infusion into the injured renal tissue. These findings from the cultured adipose MSC—atherosclerotic renal disease study are also relevant to the observations made in this study as to the degree of SVF treatment benefit seemed to be related to CKDu stage—if the kidneys are already significantly sclerotic, cell therapy is likely to be of limited benefit. An eGFR of 30 mL/minute/1.73 m^2^ was observed in this study as a potential “threshold”, determining the extent and durability of the potential therapeutic effect. The striking difference in long-term clinical response and its correlation with the GFR upon time-of-entry may reflect the extent of disease-related reductions in cellular mass and renal reserve characteristic of CKDu advanced disease.

This study constitutes the first time that fresh, non-culture expanded, adipose-derived SVF has been used for the treatment of CKDu patients. This study has a number of limitations, principally a small sample size and lack of a randomized control group. Given the fatal nature of this disease, MINSA policy precludes invasive procedures with no clear potential for patient benefit. Therefore, 12 non-SVF-treated CKDu patients were selected for a historical comparison cohort, matched for age and disease stage, as a first-pass model to compare to the SVF-treated patients and to better evaluate safety and potential clinical utility. An additional limitation was the unavailability of kidney tissue to evaluate for pathophysiological changes before and after the intervention.

Feasibility and safety were evaluated in this first-in-human study and insight was gained for future investigations as subjects with a wide spectrum of disease severity were evaluated. This study also provided evidence of potential clinical utility and for an effect size that will inform subsequent SVF clinical trials. MINSA has approved a subsequent study using a crossover design of single versus bilateral infusion such that all patients can have access to potential benefit while allowing for focus on the specific anatomic effects of SVF infusion. This follow-on study will have a larger number of patients, will concentrate on stages 3a and 3b disease, will use a standardized SVF treatment dose, and will include pre- and post-treatment histopathology.

## Conclusions

This study demonstrates the feasibility and safety of intra-arterial SVF cells as a potential therapy for the tubulointerstitial inflammatory ischemic state that characterizes CKDu. A future randomized, controlled study of SVF can offer further valuable insights for the understanding and treatment of this serious clinical problem.

## Supplementary Material

szac080_suppl_Supplementary_Table_1

szac080_suppl_Supplementary_Table_2

## Data Availability

The data that support the findings of this study are available from the corresponding author upon reasonable request.
