# Supplementary figures and images for: Safety of Stromal Vascular Fraction Cell Therapy for Chronic Kidney Disease of Unknown Cause (Mesoamerican Nephropathy)
Source: Stem Cells Transl Med. 2022 Dec 21;12(1):7–16. doi: 10.1093/stcltm/szac080 (PMC9887091; doi:10.1093/stcltm/szac080)

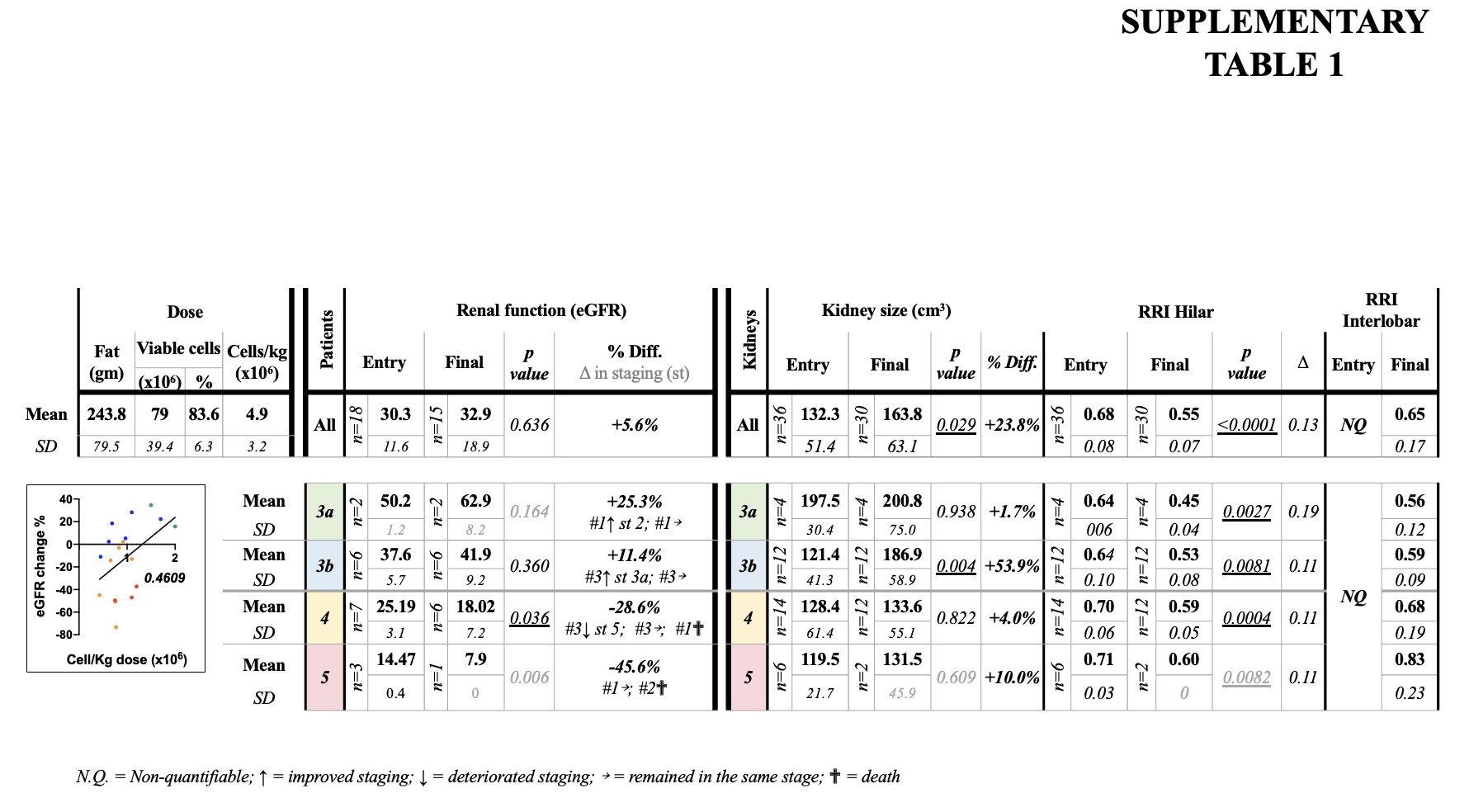

Supplement: szac080_suppl_Supplementary_Table_1 [file szac080_suppl_supplementary_table_1.jpeg]

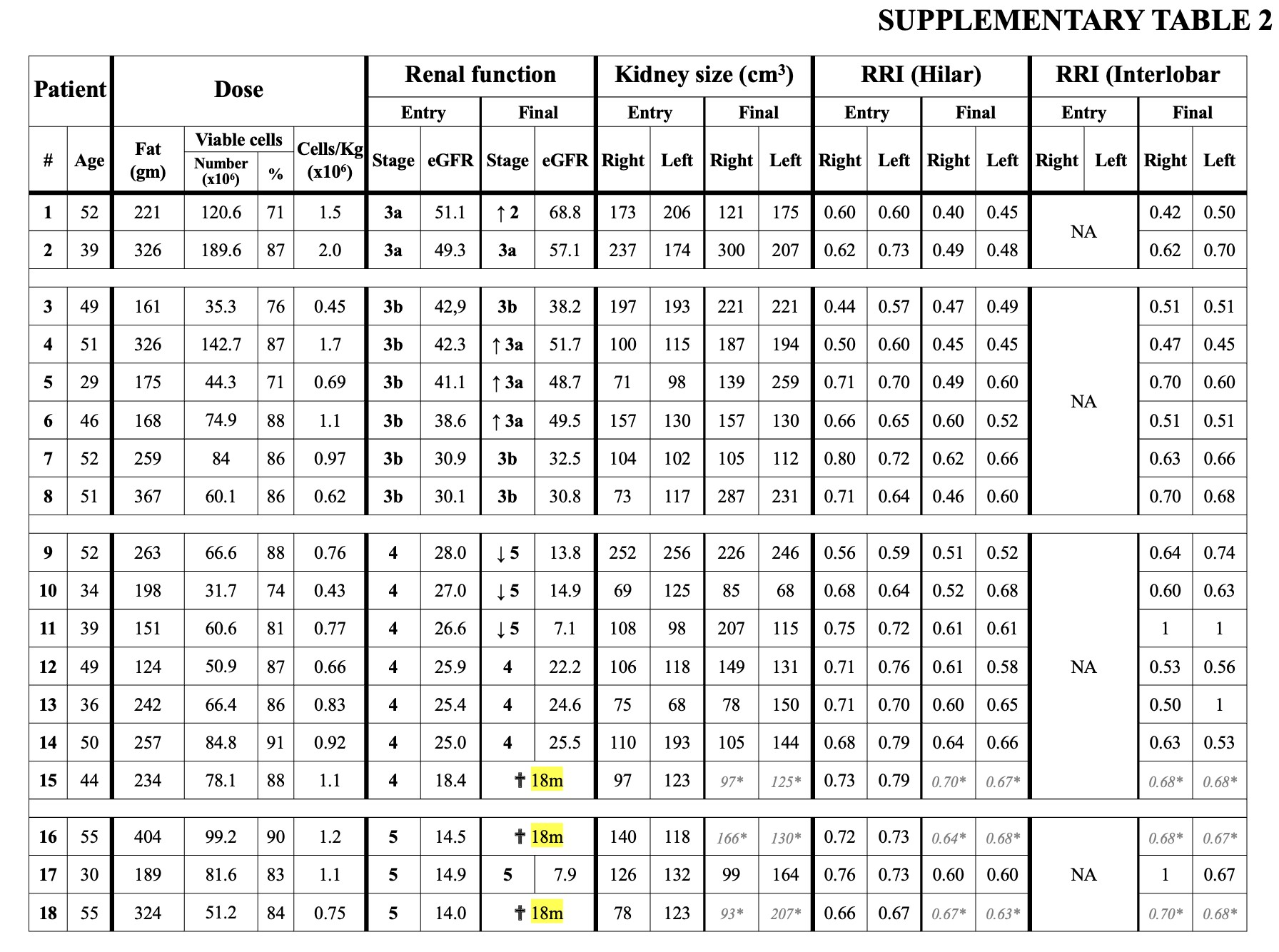

Supplement: szac080_suppl_Supplementary_Table_2 [file szac080_suppl_supplementary_table_2.jpeg]
